# Identifying Vulnerable Atherosclerotic Plaque in Rabbits Using DMSA-USPIO Enhanced Magnetic Resonance Imaging to Investigate the Effect of Atorvastatin

**DOI:** 10.1371/journal.pone.0125677

**Published:** 2015-05-14

**Authors:** Chunmei Qi, Liangrong Deng, Dongye Li, Weiheng Wu, Lei Gong, Yong Li, Qingdui Zhang, Tao Zhang, Chao Zhang, Yu Zhang

**Affiliations:** 1 General Hospital of Nanjing Military Region, Xuzhou, China; 2 Institute of Cardiovascular Disease, Xuzhou Medical College, Xuzhou, China; 3 Department of Cardiology, Second Affiliated Hospital of Xuzhou Medical College, Xuzhou, China; 4 Department of magnetic resonance imaging, Second Affiliated Hospital of Xuzhou Medical College, Xuzhou, China; 5 Laboratory of Molecular and Boimolecular Electronics, Southeast University, Nanjing, China; Brigham and Women's Hospital, Harvard Medical School, UNITED STATES

## Abstract

**Background:**

Rupture of an atherosclerotic plaque is the primary cause of acute cardiovascular and cerebrovascular syndromes. Early and non-invasive detection of vulnerable atherosclerotic plaques (VP) would be significant in preventing some aspects of these syndromes. As a new contrast agent, dimercaptosuccinic acid (DMSA) modified ultra-small super paramagnetic iron oxide (USPIO) was synthesized and used to identify VP and rupture plaque by magnetic resonance imaging (MRI).

**Methods:**

Atherosclerosis was induced in male New Zealand White rabbits by feeding a high cholesterol diet (n = 30). Group A with atherosclerosis plaque (n = 10) were controls. VP was established in groups B (n = 10) and C (n = 10) using balloon-induced endothelial injury of the abdominal aorta. Adenovirus-carrying p53 genes were injected into the aortic segments rich in plaques after 8 weeks. Group C was treated with atorvastatin for 8 weeks. Sixteen weeks later, all rabbits underwent pharmacological triggering, and imaging were taken daily for 5 d after DMSA-USPIO infusion. At the first day and before being killed, serum MMP-9, sCD40L, and other lipid indicators were measured.

**Results:**

DMSA-USPIO particles accumulated in VP and rupture plaques. Rupture plaques appeared as areas of hyper-intensity on DMSA-USPIO enhanced MRI, especially T2*-weighted sequences, with a signal strength peaking at 96 h. The group given atorvastatin showed few DMSA-USPIO particles and had lower levels of serum indicators. MMP-9 and sCD40L levels in group B were significantly higher than in the other 2 groups (P <0.05).

**Conclusion:**

After successfully establishing a VP model in rabbits, DMSA-USPIO was used to enhance MRI for clear identification of plaque inflammation and rupture. Rupture plaques were detectable in this way probably due to an activating inflammatory process. Atorvastatin reduced the inflammatory response and stabilizing VP possibly by decreasing MMP-9 and sCD40L levels.

## Introduction

Atherosclerosis (AS) is a pathological progression of chronic vascular inflammatory with a long time-course based on vascular lesions; it can cause severe complication such as myocardial infarction and stroke [1]. Therefore, early detection of vulnerable atherosclerotic plaques (VP) or rupture plaque to allow early and effective interventions is becoming a major research interest. Morphologically, a typical VP often contains a large lipid-rich athermanous core covered by a thin fibrous cap, and is infiltrated by inflammatory cells, such as macrophages [2].

Several imaging approaches have been developed to detect VP, but early identification of plaques at high risk of plaque rupturing and triggering thrombosis within a short space of time remains a challenge. Angiography is the most common technique used to assess cardiovascular risk by imaging arterial stenosis, but with the limitation that it cannot assess those lesions most prone to early rupture [3]; it also provides little information on the composition of the atherosclerotic plaque. High-resolution intravascular imaging modalities, including intravascular ultrasound virtual histology and optical coherence tomography, permit direct imaging of the plaques and vessel wall [4]. However, these methods are invasive, expense and require expertise. PET is an advanced non-invasive technique with high accuracy combining anatomical and metabolic imaging in animal [5] and human studies [6, 7], but it is expensive and is limited by ionizing radiation.

MRI, with no ionizing radiation, but with repeatability, can provide 3-dimension more stereoscopic information, and is quicker than traditional histological detection. Macrophages are important in the rupture of VP [8]. With the development of molecular imaging with ultra-small super paramagnetic iron oxide (USPIO) as the MRI contrast agent has, in particular, become a research topic of considerable interest. USPIO is a new type of MRI contrast agent that can be taken up by macrophages. USPIO particles accumulate in the shoulders or necrotic lipid cores of ruptured VP [9], and therefore it is useful for detecting macrophages in atherosclerotic plaques in murine species [10]. It has also been possible to image carotid plaque inflammation with USPIO-enhanced MRI in humans [11], which is based on stable plaques. Our research, however, is based on unstable and ruptured plaques unlike previous studies.

Considering the widespread use of dextran-coated particles in biological applications, it is surprising that the nature of the interaction between this coating and iron oxide surface has not been more extensively investigated [12]. The presence of the coating is fundamental to modulate the USPIO fate by masking and controlling its electrical surface properties [13]. We have used a new MRI contrast agent, DMSA-USPIO [14] synthesized by our laboratory. Owing to its small size and hydrophilic coating, DMSA-USPIO should be useful in studying arteriosclerotic rupture plaques.

Hyperlipidemia is a major risk factor for AS. It can make the blood flow rate slow and increase viscosity, thus making lipid more easily deposited in the vessel wall, gradually forming part of atherosclerotic plaques.

A VP model widely reported can be the successful establishment of lesions through balloon-induced vascular endothelium injury, and transfecting the abdominal aorta with the p53 gene in rabbits given a high fat diet [15, 16]. Changes of rupture plaques under pharmacological triggering is our major research interest.

Inflammation plays an integral part in the development and progression of VP, leading eventually to plaque instability. How can we evaluate inflammation in the living body? Certainly many inflammatory indicators are known, including C-reactive protein, interleukin 6, soluble CD40 ligand (sCD40L), matrix metalloproteinase-9 (MMP-9) and others. The key treatment for stabilizing VP is the inhibition of inflammation [17]. However, little is known about MMP-9 and sCD40Lk in these circumstances.

Atorvastatin, a hydroxymethylglutaryl-CoA redutase inhibitor, is one of the most widely used statins in the primary and secondary prevention for coronary heart disease. New clinical guides point out that atorvastatin has the effect of being anti-inflammatory, lowering lipid, stabling plaque and even reversing it. Atorvastatin can also reduce plaque destabilization by downregulating matrix metalloproteinase (MMP) [18] and proinflammatory cytokines [19]. The benefit of statin therapy is related not only to its lipid-lowering, but to its anti-inflammatory properties.

We established an atherosclerotic rabbit model of VP by fed high cholesterol diet combined with balloon injury in the abdominal aortic and locally injection with recombinant p53 adenovirus in the atherosclerotic plaques. We used Chinese Russell’s viper venom and histamine to trigger plaques as a result of acute plaque rupture and thrombosis model formation which can not be operated on human body. We used dimercaptosuccinic acid (DMSA) instead of dextran and its derivatives to identify plaque inflammation by the marked accumulation of DMSA-USPIO within macrophages in VP and rupture plaques. Whether atorvastatin reduces the vulnerability of rabbits to abdominal aorta injury by decreasing the levels of MMP-9 and sCD40L is unknown.

## Materials and Methods

### Animal model

Thirty male New Zealand white rabbits (mean weight 2.0–3.0kg, Animal Center of Xuzhou Medical College) were included in the study. The Principle of Laboratory Animal Care published by the National Institute of Health in 1996 was followed. Approval for the experimental protocol was granted by the Animal Care Committee of Xuzhou Medical College. The rabbits were given 1 week to adapt to their new environment and were raised separately in an air-conditioned room.

They were randomly divided into 3 groups each of 10 animals: group A, control; group B, VP model; group C, VP and atorvastatin. The rabbits were allowed water ad libitum. Group A were fed on a hyper-cholesterolemic diet (1% cholesterol, 0.2% bile salt, 8% lard, 10% yolk powder, 80.8% normal diet) of 150 g per kg per day for 8 weeks. Groups B and C had balloon-induced endothelial injury of the abdominal aorta, followed by right femoral artery ligation. In brief, the rabbits were anesthetized through the injection of 30mg/kg sodium pentobarbital via rabbit ear vein. Then the puncture of the right femoral artery was performed and a balloon catheter of 3.5mm diameter and 15mm length was introduced into the aorta about 20cm along the 0.014inch guide wire. The balloon was inflated by injecting distilled water up to 8 atmospheres and then was pulled back to the common iliac artery. The balloon was repeatedly pulled back for three times in order to injure abdominal aortic endothelium. The femoral artery was ligated and intramuscular injection of penicillin was given to prevent infection. They were also fed a hypercholesterolemic diet. After 8 weeks, group C were given atorvastatin (3.0 mg per kg per day[20]) which was mixed in the hyper-cholesterolemic diet until the end of 16 weeks. After 16 weeks, the rabbits were examined by MRI to determine if VP had formed. Abdominal aortic plaques in groups B and C were transfected with recombinant adenovirus-carrying human wild-type p53 gene [21]. Two weeks later, the rabbits were pharmacologically triggered with Chinese Russell’s viper venom 0.15mg per kg (Guangdong Research Institute of Snake Venom, Guangdong, China). The venom was injected intraperitoneally, followed by 0.02 mg per kg an injection of histamine (Dongfeng Biological Technology, Shanghai, China) in an ear vein 30 min later. At last, all the experimental rabbits were to be put to death by intravenous injection of an overdose of sodium pentobarbital through ear marginal vein.

### High-resolution multi-sequence MR imaging

MRI scanning was done with a 1.5-tesla clinical system (Gyroscan Intera 1.5T; Philips Medical System, Eindhoven, The Netherlands) before pharmacological triggering to measure the signal strength of each sequence in the plaques. The sequence parameter of MRI settings is shown in **Table 1.** USPIO (Fe_3_O_4_/DMSA, DMSA for surface modification molecules: two sulfur succinic acid, TEM electron size ~10 nm, water power size 20–30 nm, Laboratory of Molecular and Biomolecular Electronics, Southeast University, Nanjing, China) was injected at 0.1 mmol per kg in an ear vein before the rabbits were MRI scanned, which was repeated at 24, 48, 72, 96 and 120 h to follow changes in the signal-to-noise ratio (SNR) in the lesions.

**Table 1:** The sequence parameter of MRI.

	T1WI	T1WISPIR	T2*WI	PDWI	T2WI
**TR(ms)**	470	300	3000	2500	600
**TE(ms)**	5	15	85	11.5	13.4
**Roll angle**	80	-	-	-	-
**Thickness(mm)**	4	4	4	4	4
**Layer spacing(mm)**	0	0	0	0	0
**Matrix**	256×256	256×256	256×256	256×256	192×256

FSE: fast Spin echo; T1WI: T1-weighted imaging; T2WI: T2-weighted imaging; PDWI: proton density-weighted imaging; FFE T2*WI: fast field echo T2*-weighted imaging.

**Fig 1:**
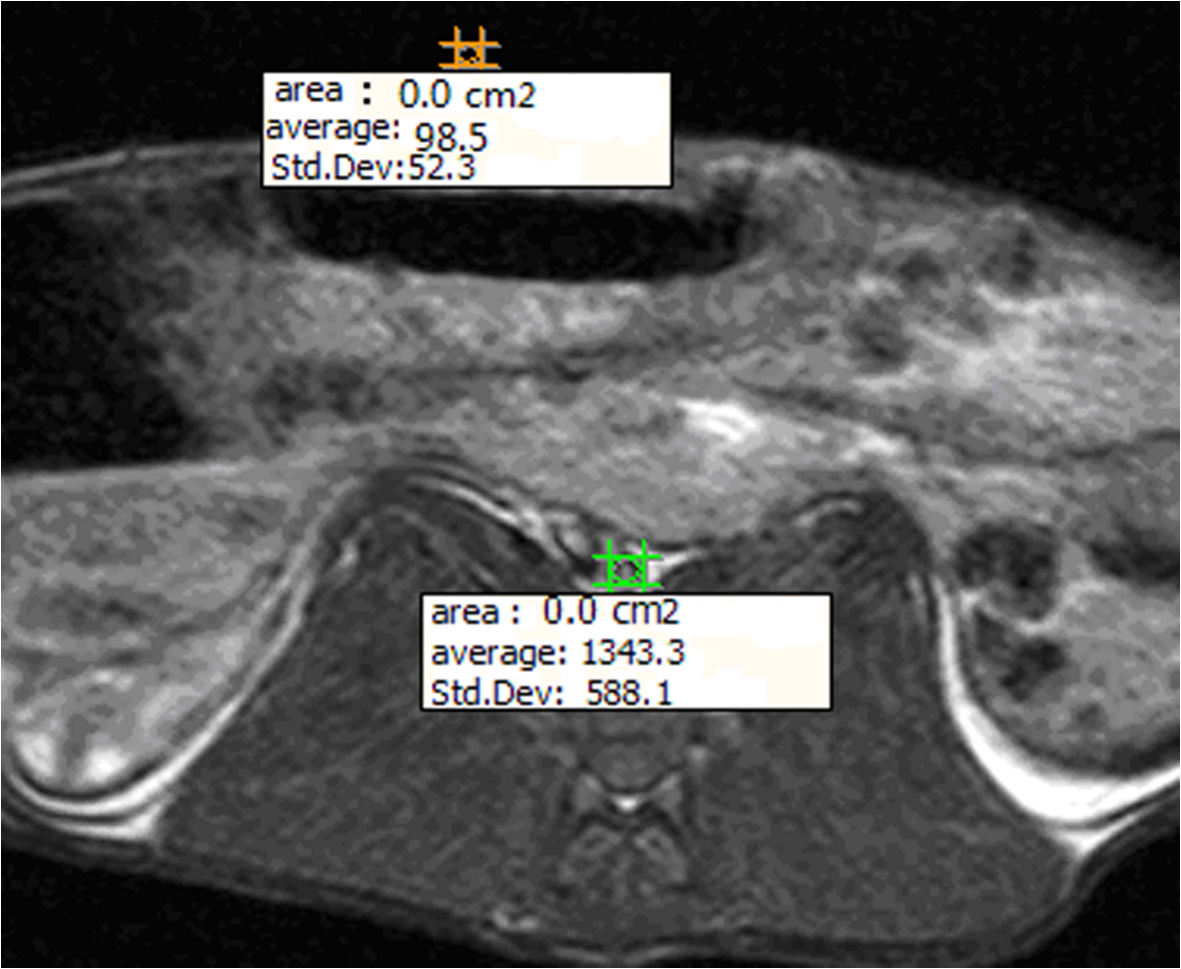
SNR on MRI. SNR was determined, where SI (signal intensity) is the average signal strength of the abdominal aorta wall, and SD is the standard deviation of the signal intensity in the background of the scan.

SNR was determined as follows(1):

SNR=SI/SD (1)

where SI (signal intensity) is the average signal strength of the abdominal aorta wall, and SD is the standard deviation of the signal intensity in the background of the scan (Fig 1). The data was used to determine the external elastic membrane area (EEMA), lumen area (LA), plaque area (PA), percent area of stenosed lumen (LAS%), vessel diameter (VD), lumen diameter (LD), plaque thickness (PT), and eccentricity index (EI). The EI is determined with the minimum and maximum PT values, as follows(2):

$$\mathrm{EI}=({\mathrm{PT}}_{\max}-{\mathrm{PT}}_{\min})/{\mathrm{PT}}_{\max}$$ (2)

When the EI is <0.5, the plaque is considered concentric, when EI is >0.5, it is eccentric.

### Collection and detection of blood samples

Before and after the rabbits were killed, serum total cholesterol (TC), triglyceride (TG), high-density lipoprotein cholesterol (HDL-C) and low-density lipoprotein cholesterol (LDL-C) were measured from mainline from ear edge vein at 0 and 16 weeks. Serum at 0 and 16 weeks was collected by centrifugation (10 min at 3000 rpm), and stored at -80°C until analysis MMP-9 and sCD40L using the appropriate ELISAs (Xi Tang Biotechnology, Shanghai, China).

### Histopathology

At necropsy, abdominal tissues were removed and washed in saline. The aorta and iliofemoral arteries were opened for inspection. Arterial tissue sections (4 μm) were obtained from the aortic segments of 25 rabbits, yielding a total of 350 segments. The abdominal aortas were excised and cut into 3 segments. One segment was placed in cold phosphate-buffered saline, cleaned of adherent connective tissue, and fixed in 4% formaldehyde for 24 h for histopathological examination. After fixation, the second segment was embedded in paraffin and cut into 5 μm sections stained with hematoxylin and eosin (H&E), Masson and Prussian blue.

### Matching the MRI Slices with Histology

The distances from the abdominal aorta and the iliac bifurcation were used as internal reference points to compare the MRI slices with histology. The aortas were marked with suture ligatures on the right artery of the iliac bifurcation over the total length imaged by MRI. The abdominal aorta was cut into 4 mm segments, and compared with the MRI slices.

### Electron Microscopy

The third segment was fixed in 3% glutaraldehyde with 1% osmium tetroxide post-fixation. They were rinsed, dehydrated and embedded in Spurr epoxy resin. After trimming, ultrathin sections (70 nm) were cut, and double stained with uranylacetate and lead citrate. The ultrastructure was examined in a transmission electron microscope (HITACI H-600, Japan) and rupture plaques photographed.

### Statistical analysis

Statistical analyses used SPSS 16.0 software (SPSS, Chicago, IL, USA). Quantitative variables are expressed as mean ± SD. Statistical comparisons were made by T-Tests and Two-way Analysis of Variance. P<0.05 was considered a statistically significant difference.

## Results

### Animal model

Animals model was successfully generated in 25 (group A, n = 9; group B, n = 8; group B, n = 8) of the 30 animals (83.3%). One animal were died of diarrhoea in group A, 2 died of plaque rupture in group B, and 2 died of diarrhoea and serious liver damage in group C.

### Magnetic Resonance Imaging

**Fig 2:**
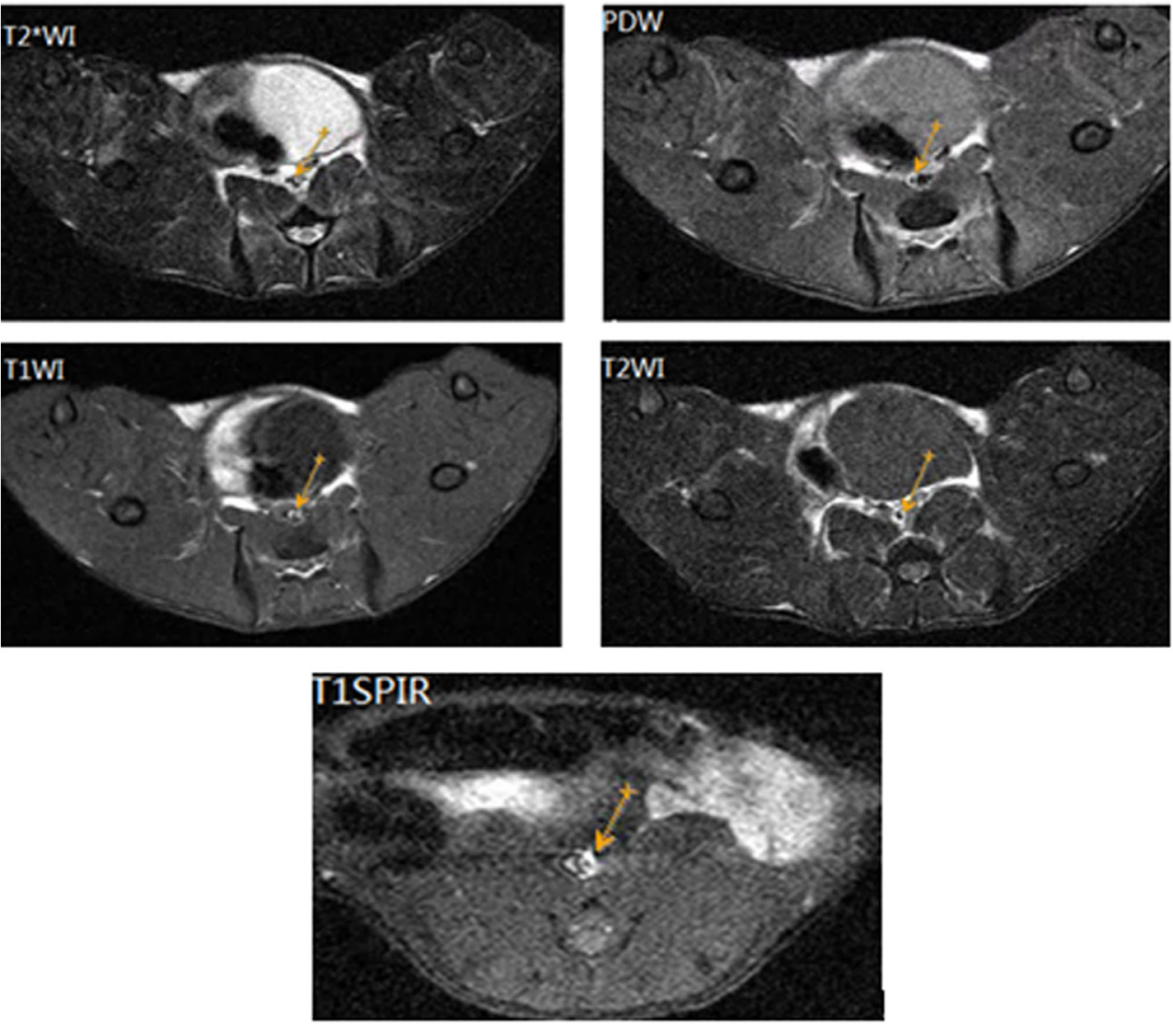
Image changing signal in multi-contrast MRI sequences of DMSA-USPIO enhanced. Image of plaques in T1WI and PDW showing a slightly higher signal and an equal signal in T2WI.

Different plaque components at MR contrasted distinctively with each other. The image of plaques in T1WI and PDWI showed a slightly higher signal and an equal signal in T2WI. A decrease of the signal strength was not obvious on spectral presaturation inversion recovery in T1WI (Fig 2). The images show characteristics of VP, such as thin or ruptured fibrous caps, large lipid or necrotic cores, and intraplaque hemorrhage (Table 2).

**Table 2:** Characteristics of plaque components and thrombus in multicontrast MRI sequences.

Plaque Component	T1WI	T2WI	PDWI	FSE-T2*WI
**Lipid-rich necrotic core**	High	Variable	High	Low
**Recent hemorrhage**	High to moderate	Variable	Variable Isointensity	Variable
**Fibrous tissue**	Moderate	Variable	Variable	Variable
**Intimal calcification**	Low	Low	Low	Variable

**Fig 3:**
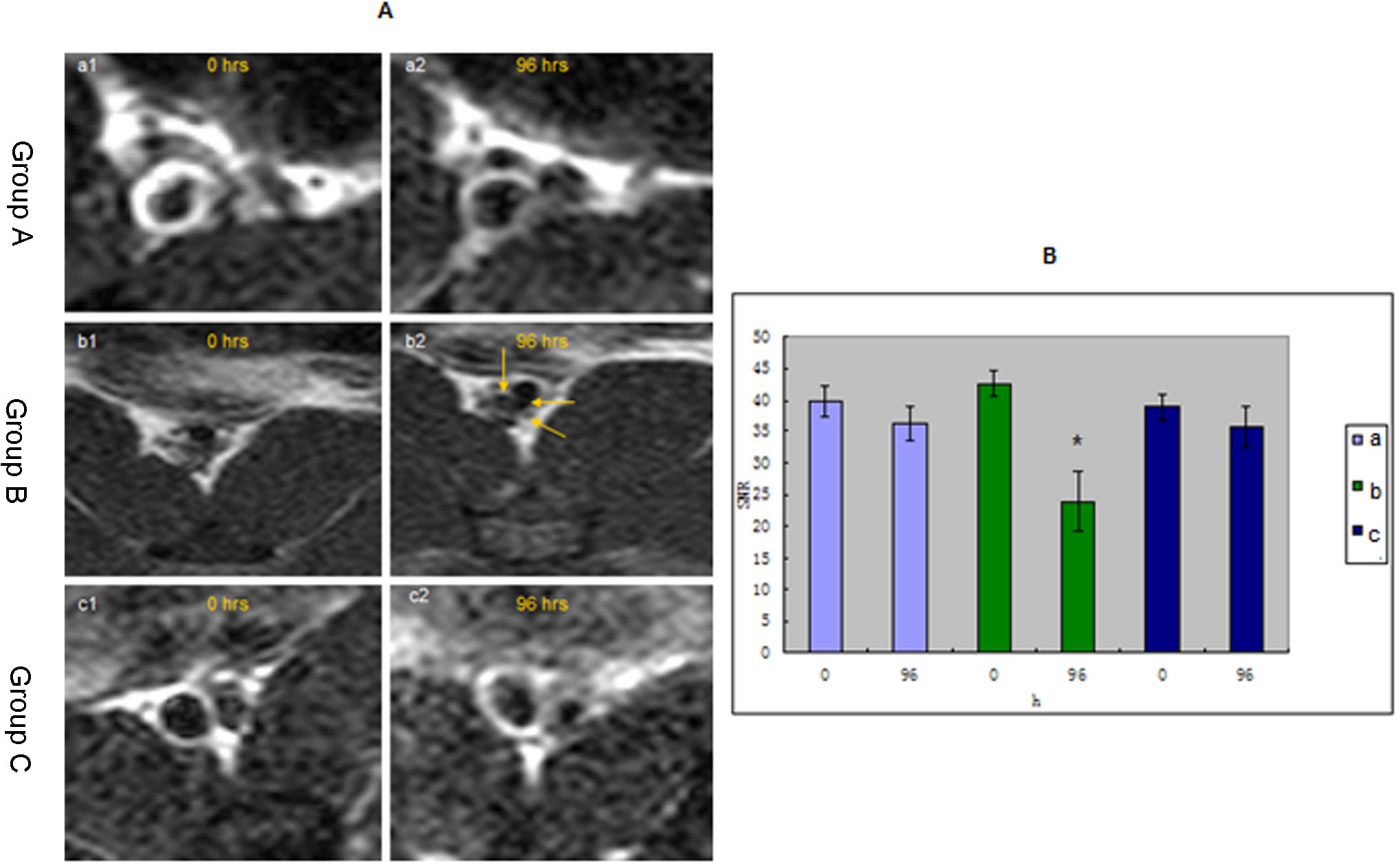
Images (A) and SNR change(B) of the 3 groups (a, b, c) between 0 and 96h after injecting USPIO. Both had signal loss at 96h compared with 0h. VP in group B (A-b) had variable changing signal. *Group B gave the peak of negative enhancement at 96 h.

**Fig 4:**
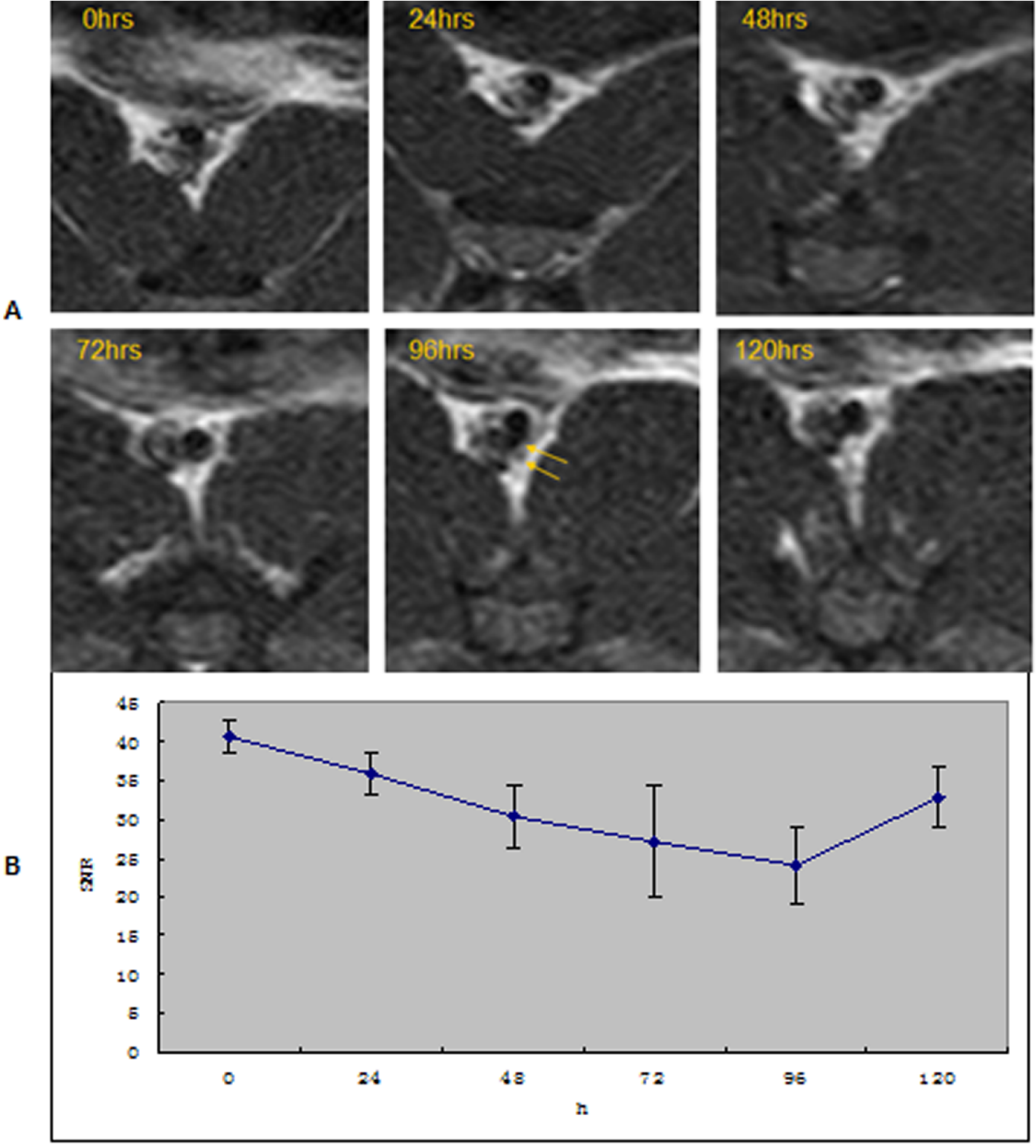
SNR trend of images (4-A) of MRI in T2*WI and chart (4-B) of group B before, and at 24, 48, 72, 96, 120h after DMSA-USPIO infusion. Maximal signal loss (yellow arrow) occurs at 96 h. The SNR trend chart shows that maximal signal loss occurs at 96 h for T2*WI in group B.

**Fig 5:**
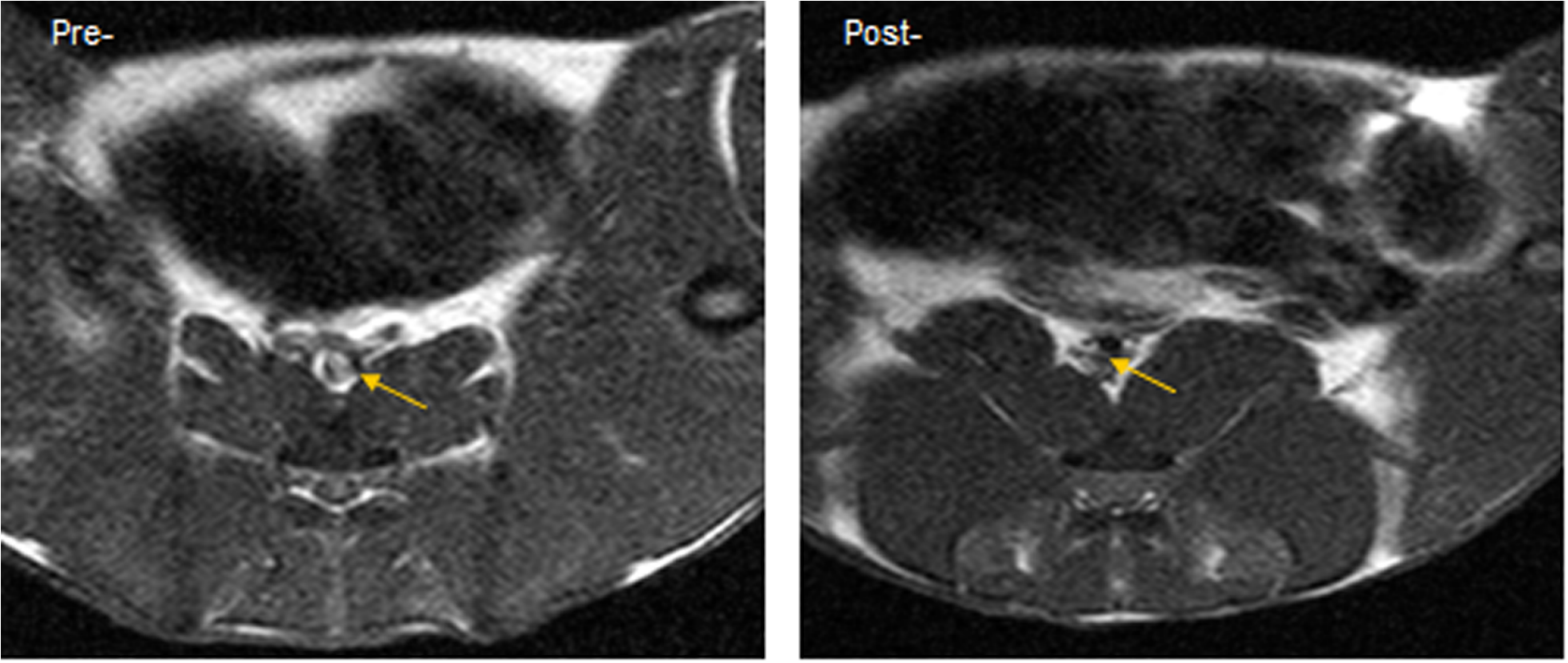
Pre and post times of rupture plaque after DMSA-USPIO infused in T2WI. Acute mixed hemorrhage showed variable signal loss of post rupture plaque compared with pre rupture plaque.

The image of plaques in T1WI and PDW shows a slightly higher signal and an equal signal in T2WI (Fig 2). There were no significant differences between groups A and C. Group A had moderately stenosed lumina, which had slight eccentric thickening. Lumen stenosis in group B was the most serious among the 3 groups (Fig 3). Post-infusion T2*WI images showed evidence of new areas of signal loss in the periluminal region with a thick fibrous cap, as seen on MRI in group B in Figs 3 and 4. The central signal of the plaques was significantly reduced after injecting USPIO, and the peak of negative enhancement occurred at 96h, especially in the T2*WI in group B (Fig 4). Acute and subacute mixed hemorrhage had a high SI in T1WI images, but only slightly high in PDWI, T2WI and T2*WI images (Figs 2 and 5). There were obvious differences between group B and the other 2 groups regarding EEMA, LA, PA, LAS%, VD, LD, PT, and EI values (Table 3).

**Table 3:** Comparision of MRI parameters for the 3 groups.

	Group A	Group B	Group C
**EEMA(mm** ^**2**^ **)**	8.39±1.58	11.95±1.62* #	9.80±0.67*
**LA(mm** ^**2**^ **)**	5.24±1.29	6.15±0.83* #	7.84±0.36*
**PA(mm** ^**2**^ **)**	3.15±0.67	5.80±1.44* #	1.03±0.15*
**LDmax(mm)**	1.16±0.16	1.59±0.15#	2.80±0.36*
**LDmin(mm)**	0.91±0.11	0.73±0.09* #	2.78±0.16*
**VDmax(mm)**	2.34±0.32	2.99±0.18* #	2.82±0.65*
**VDmin(mm)**	2.22±0.30	1.92±0.20* #	2.12±0.32*
**PTmax(mm)**	0.25±0.05	1.08±0.17* #	0.10±0.23*
**PTmin(mm)**	0.16±0.05	0.48±0.14* #	0.08±0.06*
**LAS(%)**	37.97±7.62	48.00±9.21* #	26.83±6.50*
**EI**	0.38±0.14	0.56±0.09* #	0.40±0.19*

* Group B and group C compared with group A, P <0.05

# Group B compared with group C, P <0.05

### Biochemical results

A high-cholesterol diet led as anticipated to lipid disorders. Compared with the baseline levels, the blood lipid level significantly increased in all 3 groups by 16 weeks. The level in group B were clearly higher than in group A at 16 weeks, but had decreased in group C that was given atorvastatin (Table 4). MMP-9 and sCD40L were significantly higher in groups A and B, but were lower in group C. Group B had significantly increased compared with group A by 16 weeks (Table 5).

**Table 4:** Comparison of blood lipid levels before and after the experiment.

	Group A	Group B	Group C
**TC (mmol/L)**			
**0**	1.313±0.389	1.320±0.069	1.313±0.050
**Week 16**	10.377±0.723*	11.243±0.723*	2.475±0.337* #
**LDL-C (mmol/L)**			
**0**	0.525±0.014	0.535±0.033	0.534±0.041
**Week 16**	7.132±0.180*	8.281±0.109*	2.049±0.141* #
**HDL-C (mmol/L)**			
**0**	1.033±0.110	1.117±0.070	1.045±0.084
**Week 16**	0.656±0.064	0.693±0.197	0.698±0.017
**TG (mmol/L)**			
**0**	1.184±0.015	1.085±0.024	1.100±0.167
**Week 16**	1.241 ±0.036*	4.879±0.162*	2.105±0.095* #

# compared with group B at the end of 16 weeks, P <0.01.

* Each group compared with 0 week (baseline), P <0.01.

**Table 5:** Comparison of MMP-9 and sCD40L levels before and after the experiment.

	Group A	Group B	Group C
**MMP-9 (pg/mL)**			
**0**	32.57±14.60	35.94±11.99	34.63±13.23
**Week 16**	62.26±16.52*	111.62±0.06*	44.33±8.36* #
**sCD40L (pg/mL)**			
**0**	148.83±30.81	149.42±32.72	148.98±23.64
**Week 16**	252.41±0.03*	400.12±0.04*	178.63±0.36* #

* Compared with the level at 0 week(baseline), P <0.05.

# compared with group B at the end of 16 weeks, P <0.05.

### Histopathological analysis of abdominal aorta

Histologyically, only 1 rabbit plaque was confirmed as a VP in the 9 rabbits of group A. The rest of the abdominal aortas formed thick fibrous caps and smaller lipid nuclear atherosclerotic plaques. Group B had larger lipid-core plaques but with thin fibrous caps. Post drug treatment, 6 of the 8 rabbit (75%) in group B had formed a total of 37 VPs. All in group C had stable plaques. The data indicate that the VP model had successfully induced plaque rupture by pharmacological triggering in the rabbits. Histopathological analysis can differentiate stable from vulnerable plaques.

International consensus on the integrity database will currently be defined as VPs that have a tendency to burst, are prone to the risk of thrombosis and/or progress quickly to critical lesions. Naghavi et al. [22] established the diagnostic criteria including major criteria and minor criteria for detection of VP based on the autopsy results (S1 Table). At least one of the primary criteria can indicate the greater vulnerability of plaque. There has been no definite prospective study concerning the application of minor criteria.

**Fig 6:**
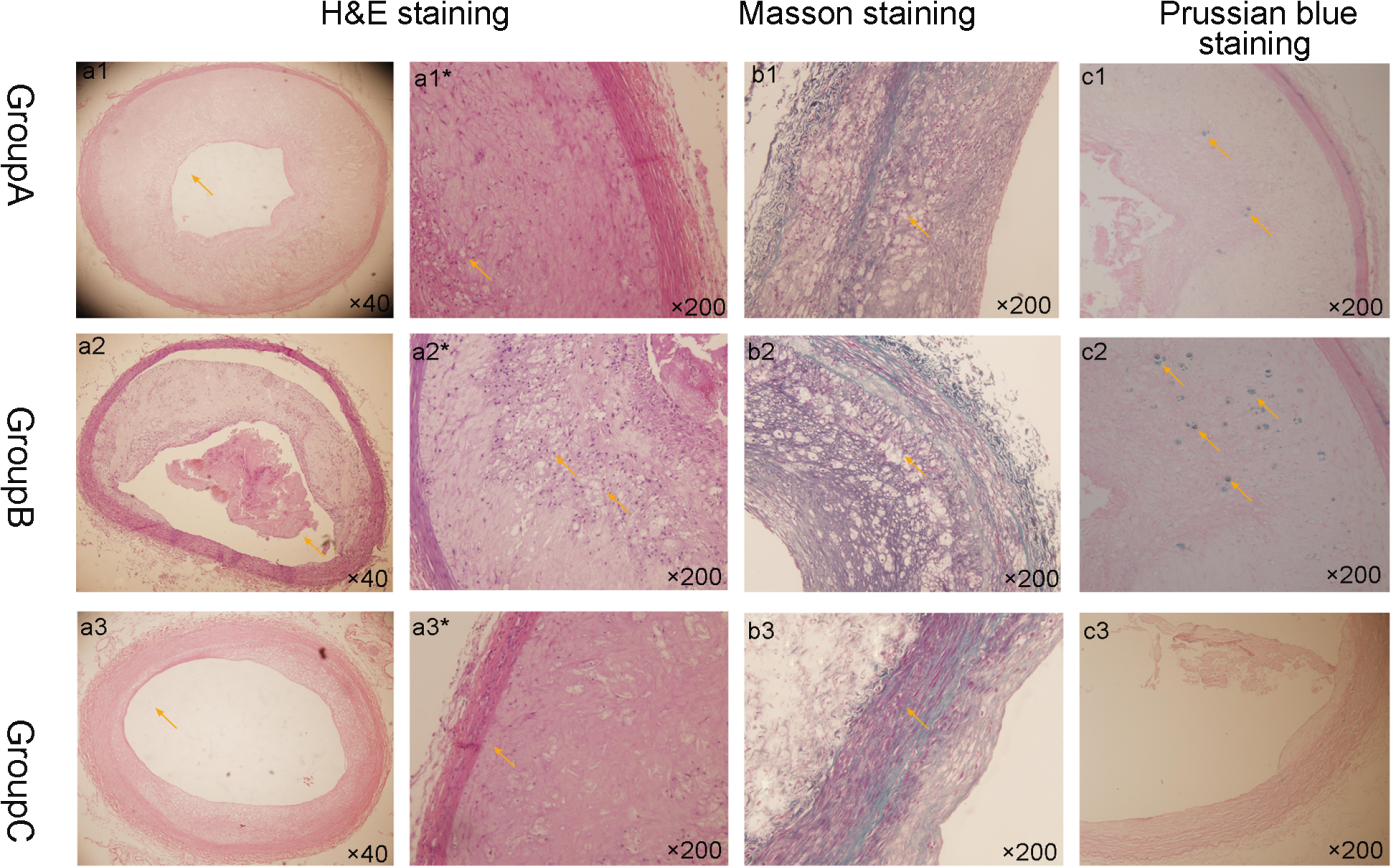
Three kinds of histology staining of sections from the 3 groups. H&E: The walls are significantly thickened in group A and B.The lumen of group C shows average thickening comparatively and the wall of plaque was thickest in group B. Intraplaque hemorrhage could be seen in group B. Large foam cells and collagen fibers are visible in group B compared with another two groups. Masson staining: hyperplasia of the collagen fibers in group B is more obvious than in another two groups. Prussian blue staining: iron particles have accumulated in macrophages in groups A and B, especially the latter; group C had very few iron particles.

A comparison of foam cells and collagen fibers among the 3 groups based on the different stains is shown in Fig 6. The wall of plaque was thickest in group B. Intraplaque hemorrhage could be seen in group B. Compared with group A, the walls in group C appeared uniform, and there is no significant increase in foam cells. Foam cells and collagen fibers were significantly hyperplastic in groups A and B, but seldom in group C. Masson stains showed hyperplasia in group B, and Prussian blue staining indicated that the iron particles had accumulated in macrophages, especially in group B (Fig 6).

### Electron microscopy

**Fig 7:**
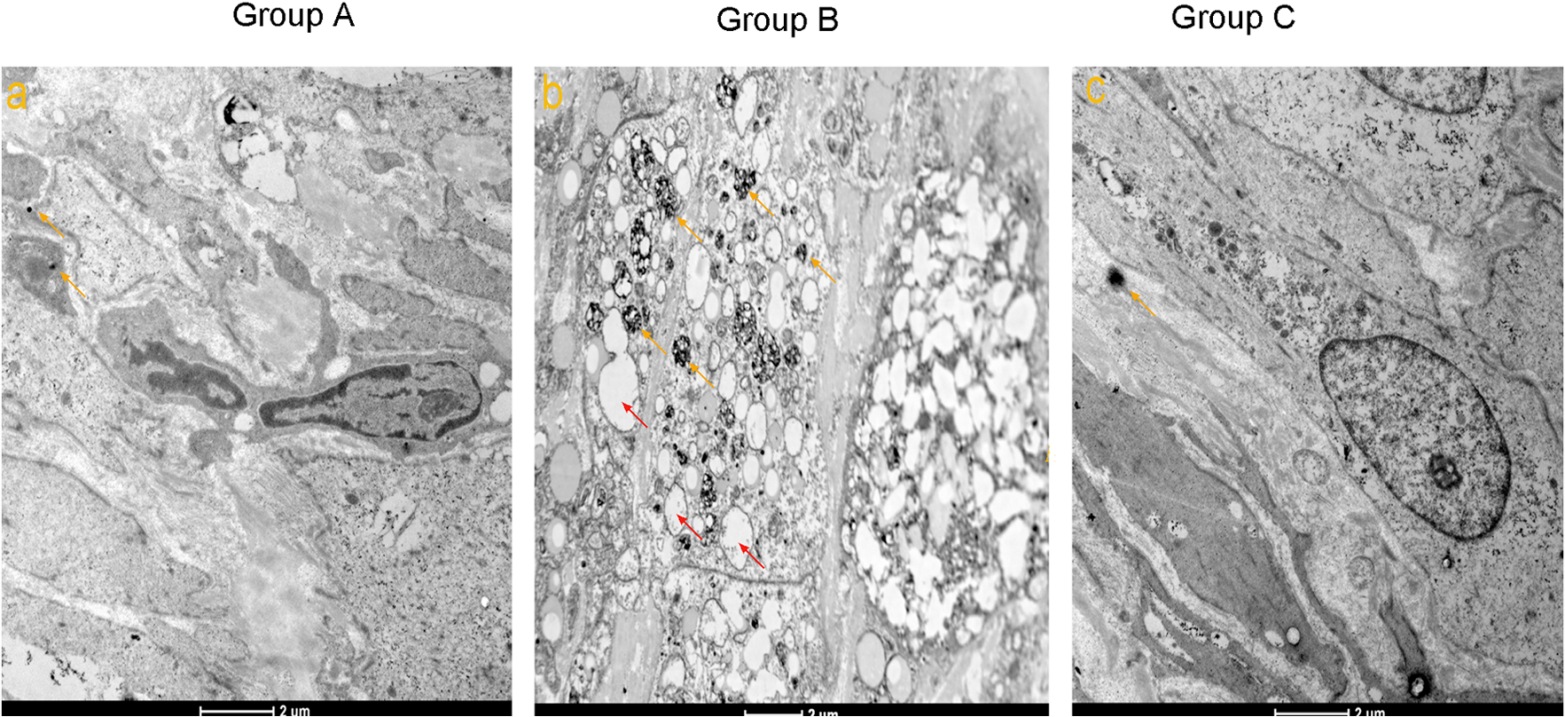
Electron microscopy of the 3 groups (a, b, c). Lysosomes and macrophages (yellow arrows) are more common in group B than groups A and C. Large fat was deposited in group B (red arrows). Iron particles (yellow arrows) are apparent in macrophages. In contrast, few Iron particles are seen in group C.

In group B (b), more lysosomes and macrophages were present than in groups A (a) and C (c). Increased fat and iron particles were apparent in the macrophages of group B (b) (Fig 7).

### SNR Results and Histopathological Analysis

**Fig 8:**
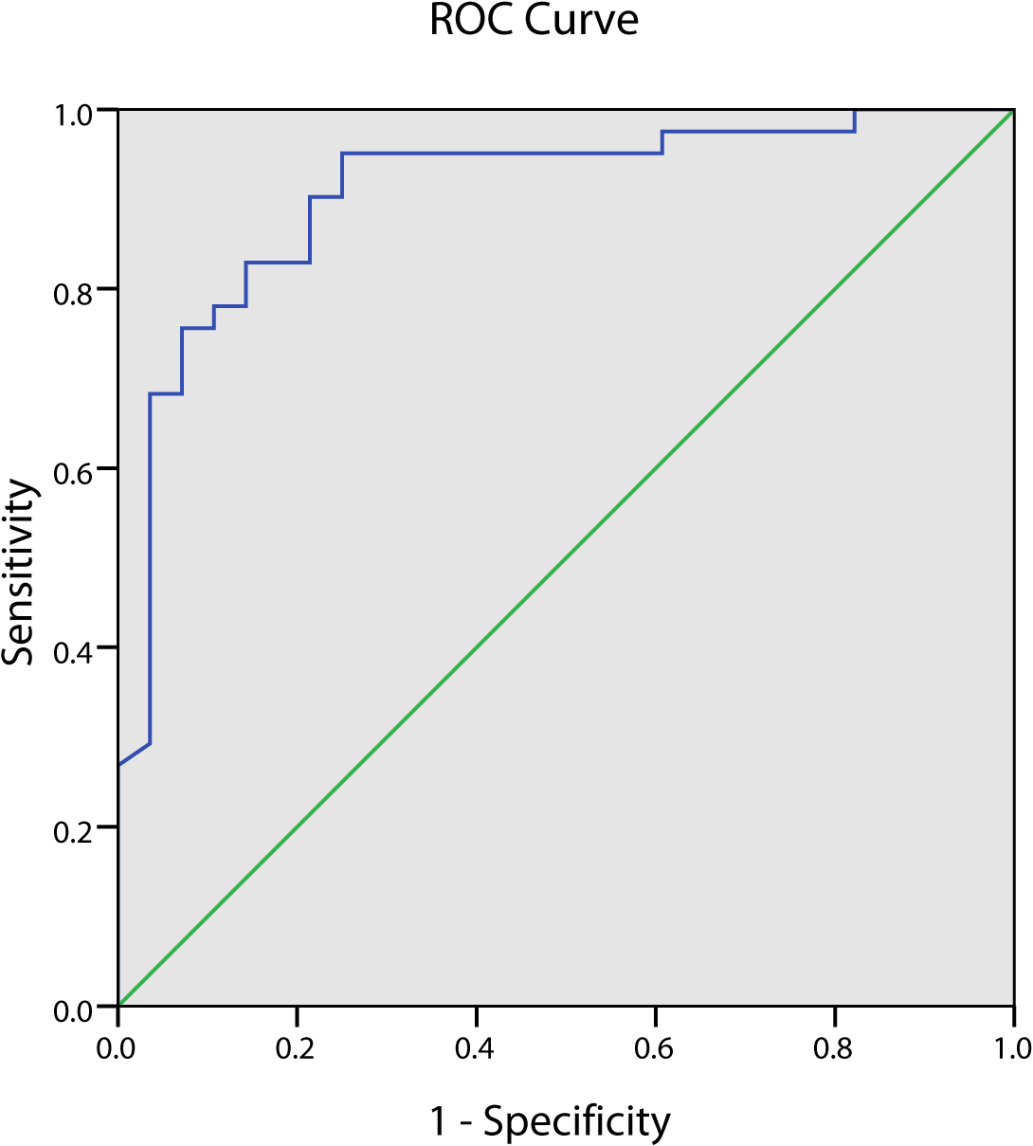
The ROC curve for SNR_mean_ to predict VP of group B at 96h. Area under the curve (AUC) was 0.908 (P = 0.000).The cutoff value was 23.9.

To measure the SNR_mean_ cutoff values predicting rupture plaque events in group B, we used the SNR_mean_ values in the arterial segments before drug triggering to plot ROC curves (Fig 8). The area under the curve (AUC) was 0.908 (95% CI = 0.835–0.981) (P = 0.0001), indicating that SNR_mean_ predicted rupture plaque events. Sensitivity and specificity were maximal when SNR_mean_ was 23.9, being 75.6% and 89.3%, respectively.

## Discussion

Early and accurate detection of VP can reduce the incidence of stroke and acute myocardial infarction. High-resolution MRI is a non-invasive technique that shows the best discrimination in soft plaque components [23, 24]. The various components, such as fat, calcification, fibrosis and thrombosis, have their own characteristic signals in MRI. Because of predominately T2 and T2* shortening effects, USPIOs are primarily used as “negative” contrast agents.

With the emergence of USPIOs that are ingested by macrophages, iron oxide as a specific magnetic contrast agent has been more widely used, such as in lymph nodes [25], nervous system diseases [26], tumor angiogenesis [27, 28], marker gene and the specific antibody detection [29]. Accumulation of USPIO in macrophages of atherosclerotic human carotid arteries plaques has been reported [30]. However, these studies concern stable plaques with severe (70%) carotid artery stenosis. Plaque triggering should not be used on human patients. We therefore used an established animal model of atherosclerotic plaque in rabbits and pharmacological triggering to induce rupture of plaques.

DMSA-USPIO is a new MRI contrast agent that was synthesized through a 2-step process [15]. In the first step, oleic acid (OA) capped Fe_3_O_4_ (OA-USPIO) were synthesized by a novel oxidation co-precipitation method in H_2_O/DMSO mixing system, where DMSO acts simultaneously as an oxidant. The second step is when OA was replaced by DMSA to obtain water-soluble nanoparticles. The mean diameter of the Fe_3_O_4_ core in DMSA-USPIO is 5.56 nm. Moreover, their ultra-small hydrodynamic size helps the particles avoid the reticulo-endothelial system and have a long half-life in circulation. As our result represented, we find much DMSA-USPIO particle accumulation in similar regions of the plaque and even rupture plaques within macrophages. DMSA-USPIO-enhanced MRI can be applied to detect macrophages in atherosclerotic plaques, and to examine the vulnerability of plaques and characteristics of plaques rupture.

Morishige et al [31] has reported that nanoparticle (MION-47) enhanced high resolution MRI can measure macrophage accumulation in cholesterol-fed New Zealand White rabbits. DMSA-USPIO (this new method) is superior to previous works. On the one hand, the diameter of the two particles between MION-47 and DMSA-USPIO is different. As all we have known, the smaller particles are more likely to be swallowed up by the macrophages. MION-47 has a ≈5-nm diameter core of superparamagnetic iron oxide coated with a ≈10-nm-thick dextran layer. In contrast, DMSA-USPIO tends to be significantly smaller and the mean diameter is about 5.56 nm as a result of more easily being intaked by macrophages. The ultra-small size helps the particles avoid the reticulo-endothelial system clean and they have a long half-life in the blood circulation. On the other hand, DMSA-USPIO specifically possesses peroxidase-like activity which MION-47 possesses not. In our previous work [15], the DMSA-USPIO were designed and synthesized by ourselves. The results have shown that the intrinsic peroxidase-like activity of DMSA-USPIO. Therefore, DMSA-USPIO was performed as the catalytic agent. Because of this feature, we are first to apply it to detect vulnerable atherosclerotic plaques. As all we know, under the effect of long-term risk factors such as hyperlipidemia, LDL-C can be accessible to vascular membrane walls through the damaged endothelium, and be oxidized into ox LDL-C. In this reacting process, DMSA-USPIO probably participates in and promotes the oxidation process because of its peroxidase-like activity as a result of synthesizing a large number of ox LDL—C and even other oxidative product. Then, macrophages can swallow more ox LDL-C through Scavenger receptor and transfer into foam cells which contain rich lipid. The more lipid contain in plaques, the more instability of plaques. Besides, as DMSA-USPIO itself can be a peroxidase-like substance, it can be more easily intaked by macrophages. As we can see in Table 2 of characteristics of plaque components in multicontrast MRI sequences, lipid-rich necrotic core showed low signal which we can distinguish on DMSA-USPIO enhanced MRI sequences. Combining these two factors, it can play a better role of enhancement on MRI. Conceivably, it can serve as a medical diagnosis regent in vivo.

PET is increasingly being used in following atheroma inflammation- a factor for high risk plaque. Measurement of ^18^F-FDG uptake by the arterial wall is a non-invasive technique that determines vascular inflammation and the risk of atherosclerotic plaque rupture [32, 33]. It is noteworthy that Khalil’s [33] study is among the few that have focused on monitoring the ^18^F-FDG uptake over time as long as 12 months. His data distinguishes ^18^F-FDG uptake in calcified and non-calcified segments of the arteries. PET may provide invaluable information regarding the cellular, metabolic and molecular composition of these plaques, and thus distinguish vulnerable from stable plaques. Quanming Zhao [31] used PET to measure the SUV_mean_ cutoff values for predicting thrombosis events; the sensitivity and specificity were 75.4% and 88.5%, respectively, compared with our SNR_means_ of 75.6% and 89.3%, respectively. Because of the high cost and ionizing radiation detrimental to human health, PET may be less suitable than DMSA-USPIO enhanced MRI.

Many researchers agree that the expression of MMP-9 increases in plaque shoulders, regions of foam cell accumulation and microvasculature. Macrophages can also secrete and synthesize proteases, such as catheptic enzyme and MMPs. Deguchiet [34] showed by using an MMP-activatable near-infrared fluorochrome that MMP activity increased in areas of the plaque with high macrophage content. The role for sCD40L interactions in atherothrombosis, in the immune response to pathogens and in thrombosis is now widely accepted. It is also deemed to be important in several inflammatory processes inducing VP. Expression of sCD40L may also be related to MMP-9 expression in atherosclerotic tissues [35]. CD40L will probably be a potential marker of instability of plaques [36]. This leads to an increase of the mediators involved in the development of atherosclerosis. High concentration of serum MMP-9 and sCD40L based on the rabbit VP model before or after treatment showed the high active inflammatory in AS in our work.

Atorvastatin has the effect of lowering lipid levels. It is one of the most widely used statins in the primary and secondary prevention of coronary artery heart disease. Atorvastatin may exert anti-inflammatory effects on phospholipases that contribute to its therapeutic benefit after acute coronary syndrome. Combined with the levels of serum inflammatory indicators, we may infer that this is the most likely mechanism of atorvastatin^’^s effect. Atorvastatin significantly reduces sCD40L and MMP-9 concentrations and activity by inhibiting VP progression, and enhances the stability of atherosclerotic plaques as seen from the histopathology of the abdominal aorta.

### Study limitations

Several limitations need to be addressed before the current imaging approach can be used in patients with atherosclerosis to follow plaque inflammation with time and the efficacy of treatments aimed at plaque stabilization. First, we need to determine whether there is an influence of USPIO long-term persistence (of organisms) in the circulation. The exact metabolic and excretion processes need to be more fully determined. Its toxicity and sensitization testing also need attention. From a technical standpoint, image resolution needs to be improved due to factors such as MRI gauge field strength and coil affecting it.

## Conclusions

We have successfully established the VP model in rabbits with the application of balloon injury to the vascular endothelium, feeding a high-fat diet, and locally transfection with exogenous recombinant wild-type P53 gene. MDSA-USPIO particles can be ingested by most macrophages. MDSA-USPIO enhanced MRI scanning provides a new method for the early detection of VP and even rupture plaques, and has high specificity and sensitivity. Atorvastatin can transform unstable into stable plaques possibly by not only decreasing the blood lipid levels, but by decreasing MMP-9 and sCD40L expression.

## Supporting Information

S1 Table Criterias for detection of vulnerable atherosclerotic plaques. At least one of the major criterias that can indicate the high vulnerability of plaque.
(DOC)
