# Supplementary material for: Identifying Vulnerable Atherosclerotic Plaque in Rabbits Using DMSA-USPIO Enhanced Magnetic Resonance Imaging to Investigate the Effect of Atorvastatin
Source: PLoS One. 2015 May 14;10(5):e0125677. doi: 10.1371/journal.pone.0125677 (PMC4431872; doi:10.1371/journal.pone.0125677)
Supplement: S1 Table — At least one of the major criterias that can indicate the high vulnerability of plaque. (DOC) [file pone.0125677.s001.doc]

Supporting information

**Table S1 Criterias for detection of atherosclerotic plaques.**

| **Major criteria** | **Minor criteria** |
| --- | --- |
| Active inflammation (Monocyte/macrophage, and T cell infiltration) | Superficial calcified nodules |
| A thin cap with a large lipid core | Yellow color (on angioscopy) |
| Endothelial denudation with superficial platelet aggregation | Intraplaque hemorrhage |
| Fissured/Injured plaque | Endothelial dysfunction |
| Severe stenosis | Expansive (positive) remodeling |
